# Screening of Protein Related to Wool Development and Fineness in Gansu Alpine Fine-Wool Sheep

**DOI:** 10.3390/ani15172578

**Published:** 2025-09-02

**Authors:** Zhaohua He, Liming Tian, Guan Wang, Fangfang Zhao, Pengfei Zhao, Shuhong Zhang, Shaobin Li, Guangli Yang

**Affiliations:** 1 College of Biology and Food, Shangqiu Normal University, Shangqiu 476000, China; hezh1250148718@163.com (Z.H.); 13680740a@sina.com (G.W.); shuhongzhang_2013@163.com (S.Z.); 2 Gansu Key Laboratory of Herbivorous Animal Biotechnology, College of Animal Science and Technology, International Wool Research Institute, Gansu Agricultural University, Lanzhou 730070, China; liming202506@163.com (L.T.); zhaofangfang@gsau.edu.cn (F.Z.); 3 Faculty of Chemistry and Life Sciences, Gansu Minzu Normal University, Hezuo 747000, China; 0700090@gnun.edu.cn

**Keywords:** wool, fineness regulation, Gansu alpine fine-wool sheep, Astral-DIA proteomics, hair follicle development

## Abstract

Fiber fineness is a critical determinant of wool quality and is of great significance in enhancing the overall quality of wool. The aim of this research was to pinpoint the key proteins that participate in the regulation of wool fineness. To achieve this, we utilized Astral—DIA proteomics technology to examine the disparities in proteins, pathways, and GO terms among the wool tissues of Gansu alpine fine-wool sheep with varying mean fiber diameters (MFD). The experiment was divided into two groups: coarse (group C, MFD = 22.36 ± 0.75 μm, *n* = 4) and fine (group F, MFD = 16.89 ± 0.36 μm, *n* = 4). The results indicated that 67 differentially expressed proteins (DEPs) were identified from the wool tissues of Gansu alpine fine-wool sheep in groups C and F. Functional enrichment analysis demonstrated that several key differential proteins, including MGST3, KRT26, KRT72, KRT74, KRT71, etc., were mainly enriched in multiple functional pathways. These pathways included glutathione metabolism, oxidative phosphorylation, the degradation of valine, leucine, and isoleucine, intermediate filaments, serine protease activity, and cysteine protease activity (*p* < 0.05). Furthermore, protein–protein interaction (PPI) network analysis suggested that type II keratin and type I keratin (such as CTSF, PSAP, TMEM106B, LYPD3, KRT71, KRT72), along with glutathione metabolism (MGST3, W5QDB7), are closely related to hair follicle development and the regulation of wool fineness. In summary, this study enriches the existing sheep proteinome database and offers novel perspectives on the regulatory mechanisms of wool fineness.

## Simple Summary

Wool quality is significantly influenced by fiber fineness, which plays a crucial role in improving the overall quality of wool. The objective of this study was to identify the key proteins involved in the regulation of wool fineness. Functional enrichment analysis indicated that several important differential proteins, such as MGST3, KRT26, KRT72, KRT74, and KRT71, were primarily enriched in multiple functional pathways. These pathways included glutathione metabolism; oxidative phosphorylation; the degradation of valine, leucine, and isoleucine; intermediate filaments; etc. (*p* < 0.05). Moreover, protein–protein interaction (PPI) network analysis revealed that type II keratin and type I keratin (e.g., CTSF, PSAP, TMEM106B, LYPD3, KRT71, KRT72), as well as glutathione metabolism (MGST3, W5QDB7), are closely associated with hair follicle development and the regulation of wool fineness. In conclusion, this research enriches the existing sheep proteinome database and provides new insights into the regulatory mechanisms of wool fineness.

## 1. Introduction

Gansu alpine fine-wool sheep is the first alpine-type fine-wool sheep breed selected and bred in China in 1981, with excellent wool production performance. This breed is primarily distributed in the alpine pasture areas of Sunan Yugu Autonomous County and Tianzhu Tibetan Autonomous County in Gansu Province, and it also generates substantial economic income for local herders [1]. In the high-altitude cold pastures of the Qinghai–Tibet Plateau and Qilian Mountains, the Gansu alpine fine-wool sheep is of inestimable economic value and ecological significance. This breed of sheep has a medium-sized body with a well-balanced structure. It is endowed with a series of remarkable traits, including cold tolerance, the capacity to grow well on coarse forage, high stress resistance, pure-white wool, and excellent climbing and moving abilities. These characteristics allow the Gansu alpine fine-wool sheep to be well-adapted to the local high-altitude ecological environment. In addition, Wool, as a natural renewable resource, has found applications in diverse industries. For instance, it is used in the textile industry as a textile raw material, in the construction industry as acoustic cotton, and in the cosmetic industry as lanolin. These applications are due to its softness, heat-insulating properties, and excellent elasticity [2]. Fineness, being one of the crucial factors affecting wool quality, plays a vital part in enhancing the overall quality of wool [3]. Finer wool generally commands a higher economic value [4]. Therefore, improving the wool fineness characteristics of Gansu alpine fine-wool sheep is of great significance for improving the wool quality of this breed, and high-quality wool products also help to improve the income of local herdsmen.

Proteomics, a well-established technological tool, is currently extensively utilized in diverse biological studies. The samples employed in proteomics could be generally divided into two main categories: tissue organs (such as the brain, heart, skin, liver, and muscle) and biological fluids (such as urine, plasma, serum, saliva, and cerebrospinal fluid) [5,6]. Previous investigations have indicated that protein recognition and protein separation technologies are crucial factors restricting the advancement of proteomics [7]. In the early phases, low-throughput approaches such two-dimensional gel electrophoresis (2-DE) was employed to separate and identify proteins in biological samples. This method is marked by its low cost and high stability [8]. At present, liquid chromatography-tandem mass spectrometry (LC-MS) has gradually emerged as the mainstream method in proteomics research. When compared with 2-DE technology, LC-MS could provide high efficiency, high sensitivity, and a wide detection range for identification and analysis [9]. Simultaneously, protein quantification techniques have also witnessed rapid development. The current mainstream protein quantification techniques mainly consist of label-free quantification, labeled quantification (iTRAQ/TMT), and mass spectrometry scanning modes [10]. Mass spectrometry scanning modes can be classified into data-dependent acquisition (DDA) and data-independent acquisition (DIA) [11]. Among these two data acquisition modes, the DDA mode has shortcomings such as poor reproducibility, data loss, inaccurate quantification, and a low detection rate for low-abundance proteins in comparison to the DIA mode [12]. Conversely, the DIA mode could offer high resolution, high throughput, and good reproducibility. Its principle is that the mass spectrometer continuously scans all ion fragments within a certain mass-to-charge ratio range to acquire more comprehensive mass spectrometry data [13,14].

At present, proteomics technology has been widely applied in biological processes related to the development of skin, hair follicles and wool. For instance, Li et al., respectively, identified 174, 157, and 156 proteins from the cashmere, wool, and yak hair. They also counted the common and unique proteins among wool samples from different breeds [15]. Moreover, Yue et al. carried out DIA proteome sequencing on the skin tissues of Alpine merino sheep with varying wool fineness. They screened out seven key proteins that might regulate the wool fiber diameter, including CIDEA, CRYM, TPST2, GPD1, and GOPC [16]. In addition, Zhao et al. conducted proteomics analysis on the skin tissues of Tibetan cashmere goats with different cashmere fineness. They discovered that VTN, AEBP1, and GPR142 could be the key candidate proteins for regulating the fineness of Tibetan cashmere goats [17]. Meanwhile, Li et al. carried out iTRAQ proteomic analysis on the wool tissues of sheep and goat breeds like Chinese merino sheep, cashmere goat, and small-tailed Han sheep. They discovered that the protein abundances of KRTAP11-1, KRT6A, and KRT38 differed significantly among various wool fibers [18]. Although certain proteins have been identified in specific sheep and goat breeds, there remains a large quantity of highly tissue-specific proteins that require further exploration. Moreover, the functions of the relevant proteins in the hair follicle development and fineness regulation of Gansu alpine fine-wool sheep are still unclear. To address this knowledge gap, we investigated the expression profiles of proteins in the wool tissues of Gansu alpine fine wool sheep.

In the present study, Gansu alpine fine-wool sheep with varying wool fineness were selected as the research subjects. By utilizing Astral-DIA proteomic technology, we analyzed the differentially expressed proteins among different wool fineness. Subsequently, functional annotation analysis was conducted, and a regulatory network was constructed. In addition, in combination with the previous data, we investigated the correlation between the key proteins and skin follicle tissue parameters. The aim was to screen the key regulatory proteins for wool fineness traits. This research is expected to contribute to the understanding of the regulatory mechanism of wool fineness in Gansu alpine fine-wool sheep. Moreover, it will offer a reference basis for the breeding of fine-wool sheep in the later stage.

## 2. Materials and Methods

### 2.1. Ethics Statement

Animal experiment was obtained approval from the Ethics Committee of Gansu Agricultural University (experimental protocol code: GSAU-ETH-AST-2021-028).

### 2.2. Experimental Animals and Sample Collection

In this study, Gansu alpine fine-wool sheep grown and bred in Songshan Township, Tianzhu Tibetan Autonomous County, Gansu Province, were used as the research observations. This area is part of the core production region for Gansu alpine fine-wool sheep. The experimental animals primarily grazed on natural pastures and received appropriate supplementary feeding. The feeding management was relatively standardized, ensuring a more consistent nutritional level among the sheep. We obtained wool samples from the left scapula of 73 one-year-old foundation ewes in July, when the wool fibers were in the growth stage of hair follicle. To measure production traits such wool fineness, wool samples were sent to the Fiber Quality Monitoring Centre in the Inner Mongolia Autonomous Region of China for assessment. The wool samples were sorted and sampled, then washed and dried. Subsequently, the fineness of the wool from 73 fine-wool sheep was measured using an optical fiber diameter analyzer (OFDA).

**Figure 1:**
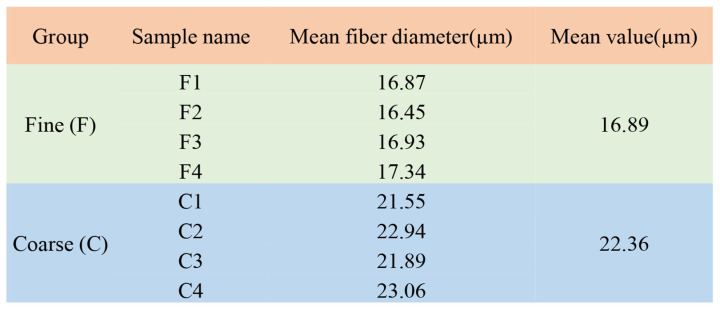
Wool mean fiber diameter measurements of experimental sheep.

In this experiment, after observing 73 Gansu alpine fine-wool sheep, according to the results of the mean fiber diameter (MFD) of the wool, 4 individuals with significant differences in wool fineness were chosen from the same group. They were then divided into a coarse wool group (group C, MFD= 22.36 ± 0.75 μm, *n* = 4) and fine wool group (group F, MFD = 16.89 ± 0.36 μm, *n* = 4) (Figure 1). These groups were used for subsequent related research. During this study, wool samples were collected from the posterior margin of the left scapula of each ewe. The wool was then washed with 1 × PBS and immediately placed in liquid nitrogen for subsequent sequencing of the wool proteome.

### 2.3. Total Protein Extraction and Mass Spectrometry Sample Preparation

Individual wool samples were first ground in liquid nitrogen. Then, they were transferred into centrifuge tubes that had been pre-cooled with liquid nitrogen. The samples were lysed using SDT (which contained 100 mM NaCl) along with an equal volume of 1/100 DTT. Subsequently, the mixture was sonicated in an ice-water bath for 5 min. After sonication, the samples were incubated at 95 °C for 8–15 min, followed by a 2 min incubation in an ice bath. Next, the samples were centrifuged at 12,000× *g* for 15 min at 4 °C. The supernatant was removed, and an appropriate amount of IAM solution (Iodoacetamide) was added at room temperature for alkylation. The alkylation process was carried out in the dark for 1 h. After alkylation, the sample was mixed with 4 times its volume of acetone (pre-cooled at −20 °C) by vertexing to ensure complete mixing. The mixture was then left to precipitate at −20 °C for 2 h. After that, it was centrifuged at 12,000× *g* for 15 min at 4 °C, and the precipitate was collected. The collected precipitate was washed with 1 mL of cold acetone. Then, an appropriate amount of dissolution buffer (DB buffer) was added to fully dissolve the protein precipitate, resulting in a protein solution. Finally, the proteins in the solution were enzymatically digested following the method reported by Buczak et al. [19]. The enzymatically digested protein filtrate was collected and lyophilized for storage.

### 2.4. DIA Mode Liquid Analysis

Prepare mobile phase A (100% water, 0.1% formic acid) and B (80% acetonitrile, 0.1% formic acid). Take 10 µL of mobile phase A liquid solution to re-dissolve the lyophilized powder. Then, centrifuge the solution at 14,000× *g* for 20 min at 4 °C. Next, inject a 200-ng sample of the supernatant into the liquid chromatography system. A Vanquish Neo Nanoscale UHPLC system (Thermo Fisher Scientific, Waltham, MA, USA) is employed. It is equipped with a C18 pre-column (174,500, 5 mm × 300 μm, 5 μm, Thermo) placed in a column oven heated to 50 °C and a C18 analytical column (ES906, PepMap TM Neo UHPLC 150 µm × 15 cm, 2 μm, Thermo). For mass spectrometry, data are collected in data-dependent mode using a Thermo orbitrap astral mass spectrometer with an Easy-spray (ESI) ion source(Thermo Fisher Scientific, Waltham, MA, USA). Set the ion spray voltage to 1.9 kV and the ion transfer tube temperature to 290 °C. The mass spectrometry data acquisition remains in data-dependent mode. The primary MS scanning range is from *m*/*z* 380 to 980, the resolution is set at 240,000 (at 200 *m*/*z*), the automatic gain control (AGC) is set to 500 %, the parent ion window size is 2-Th, the number of data-independent acquisition (DIA) windows is 300, and the normalized collision energy (NCE) is set to 25%. The secondary *m*/*z* acquisition range is from 150 to 2000, the sub-ion resolution for Astral is set to 80,000, and the maximum injection time is 3 ms. Finally, the raw data are obtained through mass spectrometry.

### 2.5. DIA Protein Data Analysis

DIA-NN software (Direct DIA, Spectronaut16) was applied to solve the raw data of spectra and comparative analysis of sheep reference genome (GCF_016772045.1_ARS-UI_Ramb_v2.0) [20]; retention time correction was applied to the iRT added in the samples, and the Q value cutoff value of the precursor ions was set to 0.01. The results of the protein quantification were subject to Differential expression statistical analyses were performed with specific screening thresholds of Fold change > 1.5 and *p*-value < 0.05.

### 2.6. Functional Enrichment Analysis

After identifying differentially expressed proteins (DEPs), they were subjected to GO (Available online: http://www.geneontology.org/ (accessed on 15 January 2025)) and KEGG (Available online: http://www.kegg.jp/kegg/ (accessed on 15 January 2025)) enrichment analyses, which in turn screened for important functional pathways and GO terms related to hair follicle development and wool fineness regulation. In addition, protein–protein interaction (PPI) analyses were performed on DEPs using STRING (Available online: http://string.embl.de/ (accessed on 20 January 2025)) for PPI analysis of DEPs, which is beneficial for revealing and discovering the key DEPs. Moreover, we used K-means clustering method for clustering analysis of proteins, which leads to further mining of protein functions.

### 2.7. Correlation Analysis

In the previous study, we measured the characteristic parameters of skin and hair follicle in coarse and fine wool sheep [21]. In the present study, we combined the relevant results of hair follicle tissue characteristic parameters determined in the previous study, Person correlation analysis was used to analyze the correlation between the key proteins and the histological characteristics of skin hair follicles and wool quality traits. Key proteins were screened based on the results of GO and KEGG enrichment analysis of DEPs in this study, and combined with previous relevant literature. * Indicates a significant correlation at the *p* < 0.05 level and ** indicates a significant correlation at the *p* < 0.01 level.

### 2.8. Statistical Analyses

Independent samples t-tests were performed on the measurements using GraphPad Prism 22.0. Results are expressed as mean ± standard deviation, with *p* < 0.05 indicating statistical significance. GraphPad Prism 8.0.1 was used for graphing.

## 3. Results

### 3.1. Data Quality Control and Protein Identification

**Figure 2:**
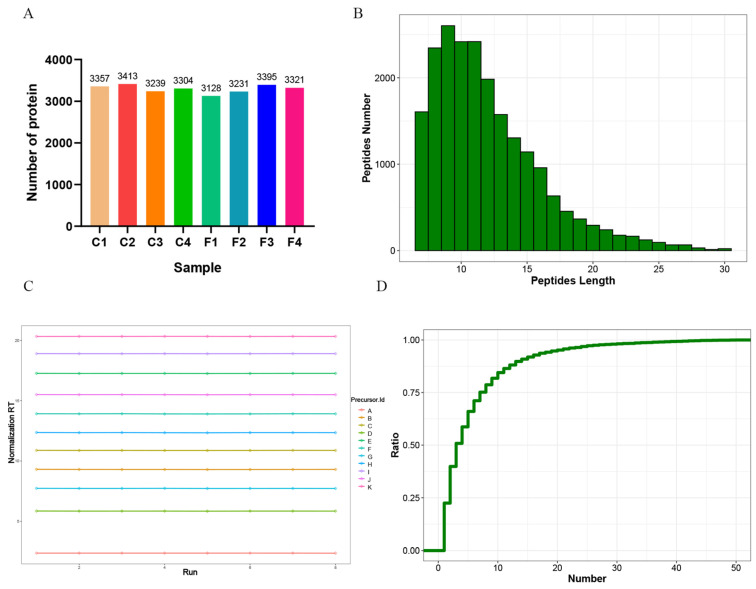
Analysis of wool protein identification results. (**A**) Statistics of the number of protein identifications; (**B**) Distribution of protein peptide length ranges; (**C**) IRT values of internal standard-corrected peptides; (**D**) Distribution plot of the number of Unique peptides in the identified proteins.

Proteins were isolated from the wool tissues of Gansu alpine fine-wool sheep in groups C and F, and their quality was examined. The results indicated that both the concentration and the total quantity of all samples satisfied the requirements for the subsequent DIA proteome sequencing experiments (Table 1). A proteomic analysis was conducted on the wool tissues of the two groups of sheep. In total of 21,106 peptides and 3558 proteins were identified (Figure 2A). Statistical analyses revealed that the peptide lengths of proteins were predominantly distributed within the range of 7–30 amino acid residues (Figure 2B), and the iRT (Indexed Retention Time) values of the internal-standard-corrected peptides were highly stable across the samples (Figure 2C). Meanwhile, the distribution curve of Unique peptides tends to be flat, indicating that the number of Unique peptides is large and more reliable proteins can be identified (Figure 2D).

### 3.2. Screening for Differentially Expressed Proteins

**Figure 3:**
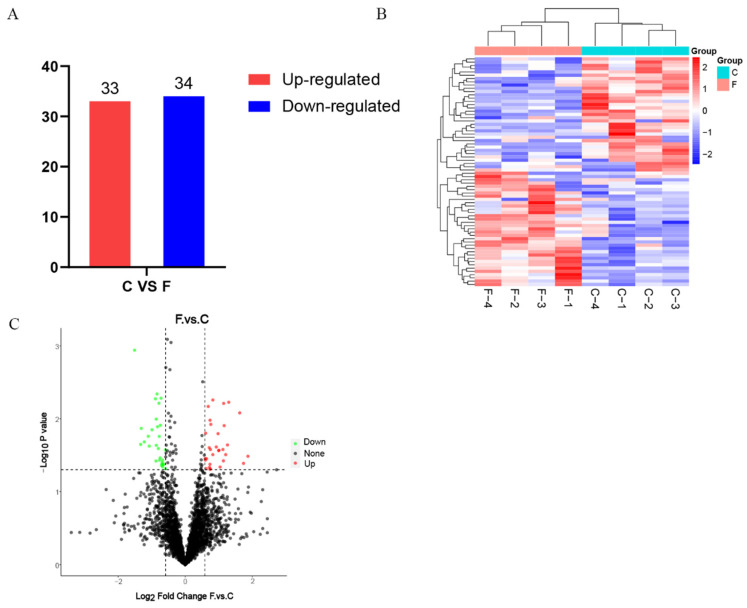
Analysis of differentially expressed proteins in wool tissues of Gansu alpine fine-wool sheep with different wool fineness. (**A**) Histogram of differentially expressed proteins of different wool fineness; (**B**) Heat map of differentially expressed proteins of different wool fineness; (**C**) Volcano map of differentially expressed proteins of different wool fineness.

In order to identify the proteins related to hair follicle development and wool fineness regulation, we compared the expression levels of the proteins identified in the wool tissues of Gansu alpine fine-wool sheep from Groups C and F. The findings showed that a total of 67 DEPs were screened in the wool tissues of the two groups of Gansu alpine fine-wool sheep. Specifically, when compared with Group C, 33 DEPs were upregulated and 34 DEPs were downregulated in the wool tissues of Group F (Figure 3A–C).

### 3.3. Functional Enrichment Analysis of Differentially Expressed Proteins

**Figure 4:**
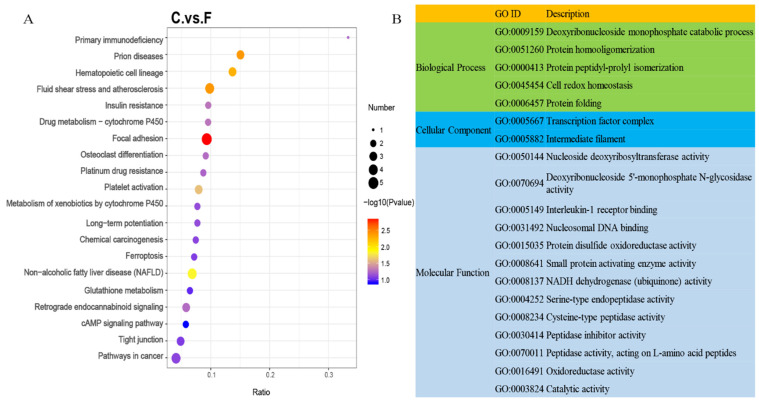
Functional enrichment analysis of differentially expressed proteins in wool tissues of Gansu alpine fine-wool sheep with different wool fineness. (**A**) Differential protein KEGG enrichment analysis; (**B**) Differential protein GO enrichment analysis.

To investigate the proteins and their function involved in the hair follicles development and fineness regulation in Gansu alpine fine-wool sheep, we carried out KEGG and GO enrichment analyses on the identified DEPs (Figure 4, Table 2). KEGG analysis results indicated that DEPs were significantly enriched in 11 pathways (*p* < 0.05), such as metabolism of xenobiotics by cytochrome P450 (ko00980) and insulin resistance (ko04931). Additionally, important pathways such as glutathione metabolism (ko00480), oxidative phosphorylation (ko00190), protein digestion and absorption (ko04974), valine, leucine and isoleucine degradation (ko00280), and cAMP signaling pathway (ko04024) were also enriched (Figure 4A). The GO enrichment analysis identified 48 GO terms with important functions, with 12, 23, and 13 terms related to cellular components, molecular functions, and biological processes, respectively. Some GO terms may be associated with hair follicle development and fineness regulation, such as protein disulfide oxidoreductase activity (GO:0015035), NADH dehydrogenase (ubiquinone) activity (GO:0008137), intermediate filaments (GO:0005882), serine-type endopeptidase activity (GO:0004252) and cysteine-type peptidase activity (GO:0008234) (Figure 4B).

### 3.4. Protein Interaction Network Analysis

**Figure 5:**
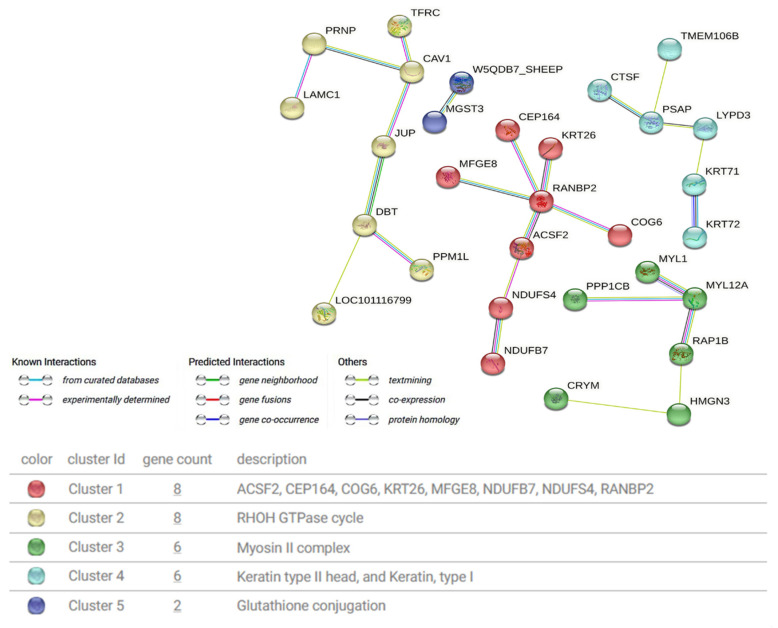
Interaction analysis of proteins related to the regulation of hair follicle development and wool fineness in Gansu alpine fine-wool sheep.

To investigate the protein interaction mechanisms related to hair follicle development and wool fineness regulation in Gansu alpine fine-wool sheep, a protein–protein interaction (PPI) network was constructed using the STRING database. K-means clustering analysis revealed that the relevant proteins could be primarily classified into five major categories, including those associated with the RHOH GTPase cycle (LAMC1, PRNP, TFRC, CAV1, JUP, DBT, PPM1L, LOC101116799), type II keratin and type I keratin (CTSF, PSAP, TMEM106B, LYPD3, KRT71, KRT72), glutathione conjugation (MGST3, W5QDB7), and other key proteins (Figure 5). Additionally, functional enrichment analysis of the proteins revealed several essential pathways and GO terms, such as intermediate filament (GO:0005882), intermediate filament protein, and Nail development (CL:25737), and Keratin, type I, and Keratin, type II (CL:25742) (Table 2).

### 3.5. Identification of Keratin and Keratin-Associated Proteins Related to Wool Fineness

**Figure 6:**
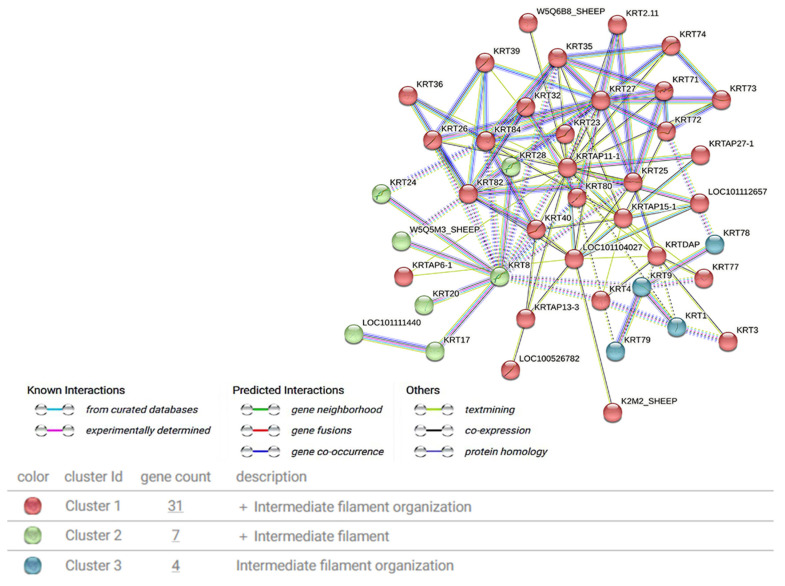
Interaction analysis of keratins and keratin-associated proteins related to hair follicle development and wool fineness in Gansu alpine fine-wool sheep.

To identify keratins and keratin-associated proteins that play a crucial role in hair follicle development and wool fineness regulation, we conducted a PPI network analysis on the 17 keratin-associated proteins and 60 keratins identified (Table 3). Clustering analysis results indicated that the relevant keratins and keratin-associated proteins primarily belong to three major categories (Figure 6). Among these, keratins such as KRT71, KRT35, KRT82, KRT26, KRT27, KRT74, and keratin-associated proteins such as KRTAP11-1 exhibit more intimate interactions. Functional enrichment analysis revealed that these keratins and keratin-associated proteins were significantly enriched in GO terms such as intermediate filament organization (GO:0045109), keratinization (GO:0031424), and epidermal development (GO:0008544).

### 3.6. Correlation Analysis Between Key Differential Proteins and Wool Fiber and Hair Follicle Characteristics

**Figure 7:**
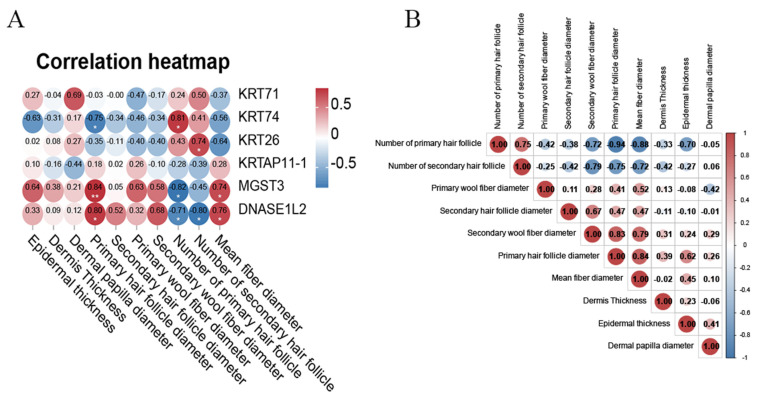
Pearson correlation analysis. (**A**) Correlation between key proteins and wool fiber and hair follicle characteristics; (**B**) Correlation between characteristics of various skin hair follicles. * Correlation is significant at *p* < 0.05, ** correlation is significant at *p* < 0.01.

To investigate the role of DEPs in regulating wool growth and fineness in Gansu alpine fine-wool sheep, Pearson correlation analysis was used to construct a correlation heatmap to explore potential relationships between key proteins and skin follicle histological characteristics (Figure 7A). In addition, we also explored the correlation between skin and hair follicle histological characteristics (Figure 7B). We found that the correlations between DEPs and wool fineness, as well as skin follicle histological parameters, were relatively abundant. Among these, KRT71, KRT74, and KRT26 showed a positive correlation with hair follicle number and a negative correlation with wool fiber diameter and hair follicle diameter, while KRT74 exhibited a negative correlation with epidermal thickness. Additionally, MGST3 and DNASE1L2 were negatively correlated with hair follicle number and positively correlated with wool fiber diameter, hair follicle diameter, and epidermal thickness. KRTAP11-1 was negatively correlated with hair follicle number and dermal papilla diameter.

## 4. Discussion

Proteomics, being a well-established technological tool, is currently extensively utilized in the research of diverse biological phenomena in both plants and animals. Moreover, proteomics research regarding the development of skin and hair follicles in sheep and goats has been documented. Guo et al. carried out proteomics research on the development of fine wool hair follicles at the embryonic stage (E87, E96, E102, E138), and they identified a total of 123 DEPs [22]. Plowman et al. conducted proteomics research on Merino wool of different coat colors (black and white). They found that certain keratin-associated proteins have an impact on sheep coat color. Specifically, proteins like KAP6, KAP6-1, KAP6-2, and KAP16-2 might be more favorable for the formation of colored wool, whereas the KAP4-3 and KAP4-7 proteins show higher expression levels in white wool [23]. The above-mentioned studies have investigated the key proteins that regulate wool and cashmere, laying a research foundation for enhancing wool traits. Nonetheless, proteomics studies related to the regulation of wool fineness in Gansu alpine fine-wool sheep are relatively rare, and as of now, no proteomics studies focusing on this particular sheep breed have been reported. In comparison to skin proteomics, employing wool tissue for proteomics analysis could more precisely identify the key proteins that influence wool growth, development, and fineness regulation. Consequently, in this study, wool tissues with different mean fiber diameters were selected for proteomics sequencing.

Protein functional enrichment analysis could more effectively screen for functional pathways that are crucial in diverse biological activities in animals. In this study, several functional pathways and GO terms were identified, which might have a role in regulating wool fineness. Including glutathione metabolism (ko04380), oxidative phosphorylation (ko00190), protein digestion and absorption (ko04974), valine, leucine and isoleucine degradation (ko00280), intermediate filaments (GO:0005882), serine-type endopeptidase activity (GO:0004252), and cysteine-type peptidase activity (GO:0008234). Related studies showed that valine, leucine, and isoleucine are the three essential amino acids for mammalian growth and are also major constituents of most proteins. Alterations in these branched-chain amino acids may lead to the development of various common diseases [24,25,26]. Moreover, proteases are significant regulators of stress responses in both plants and animals. They could play a vital part in protein degradation during protein turnover [27]. Serine endopeptidase activity is related to proteolytic activity [28], and cysteine proteases are associated with developmental and stress-induced functional patterns [29]. Furthermore, in the gene functional enrichment analysis of the fine-wool sheep skin transcriptome, significant enrichment was observed in functional pathways such as glutathione metabolism, oxidative phosphorylation, and intermediate filaments, further highlighting the critical roles of these pathways in hair follicle development and fineness regulation.

The construction of PPI network could effectively identify key proteins and functional pathways, thus offering a more comprehensive comprehension of protein function. In this research, we built a PPI network for the selected DEPs. By means of protein clustering and functional analysis, key protein molecules related to the RHOH GTPase cycle (such as LAMC1 and TFRC), type II keratin and type I keratin (including CTSF, LYPD3, KRT71, and KRT72), and glutathione conjugation (like MGST3, W5QDB7) were recognized. GTPases are a highly conserved group of regulatory proteases participating in the biological process of hydrolyzing GTP to GDP. This process is linked to protein activity and acts as a molecular switch in cellular signaling pathways [30,31]. Relevant studies have discovered that the deficiency of laminin subunit γ1 (LAMC1) might influence the morphogenesis of hair follicles. Moreover, the regulation of the BMP-MSX2-HOXC13-FOXN1 signaling axis by this protein contributes to the normal differentiation of animal hair shafts [32]. Cathepsin F (CTSF) is associated with skin aging and serves as a potential specific marker for fibroblasts and keratinocytes in aged skin [33,34]. Hence, it is hypothesized that this protein also has a regulatory function in skin and wool fiber formation. In addition, microsomal glutathione S-transferase 3 (MGST3), as a member of the glutathione transferase family, promotes the production of the lipid inflammatory mediator leukotriene C and shows glutathione peroxidase activity that depends on lipid hydroperoxides [35]. Related research has found that glutathione peroxidase is associated with the degradation of melanin in the skin [36]. Since the formation of white wool is also related to melanin degradation, microsomal glutathione S-transferase 3 (MGST3) may play a part in the formation of wool color.

Related research has demonstrated that keratin and keratin-associated proteins could play a significant regulatory part in skin follicle development and wool formation [37]. In the present study, we built a PPI network for all the keratins and keratin-associated proteins screened from the wool proteome, and 42 key regulatory factors were identified. Among them, keratins like KRT71, KRT35, KRT82, KRT26, KRT27, KRT74, and KRTAP11-1, along with keratin-associated proteins, exhibit more intimate interactions. Grilz-Seger et al. discovered that KRT27 might be related to hair follicle morphogenesis [38]. Moreover, as an inner root sheath gene, mutations in this gene disrupt the assembly of keratin intermediate filaments, thus influencing hair formation [39,40]. Base mutations in *KRT74* may participate in regulating the mechanism of human autosomal dominant alopecia [41]. The KRT71 protein is mainly expressed in the inner root sheath of hair follicles [42] and is strongly linked to curl traits in animal hair [43]. Zheng et al. observed that the *KRT35* gene could affect cashmere fineness [44]. In addition, relevant studies suggested that KRT82, being a risk gene for alopecia areata [45], is also associated with hair fiber fineness [46]. Gong et al. found that mutations in the *KRTAP11-1* gene may cause changes in gene expression, protein structure, and/or post-translational modifications, thereby affecting wool fiber structure and other wool characteristics [47]. Furthermore, in this research, the selected key proteins were notably enriched in GO terms such as intermediate filament organization (GO:0045109), keratinization (GO:0031424), and epidermal development (GO:0008544). This further emphasizes the vital role of keratin and associated protein-related regulatory factors in the regulation of wool fineness.

Conducting a correlation analysis between proteins and the skin hair follicle tissue of fine-wool sheep could facilitate a more in-depth exploration of the relationship between them, thereby providing offer more effective references for molecular marker-assisted selection in the production of fine-wool sheep. The correlation analysis results indicated that these key proteins are significantly correlated with the parameters of the skin hair follicle tissue. For instance, KRT71, KRT74, and KRT26 are positively correlated with the hair follicle count. At the same time, they are negatively correlated with the wool fiber diameter and the hair follicle diameter. Moreover, relevant studies have discovered that mutations in the *KRT71* and *KRT74* genes might result in the symptoms of Hypotrichosis simplex (HS) and wooly hair (WH) [41]. KRT71 knockout (KO) mice display a distinct curly hair phenotype and a novel phenomenon of complete hair loss within 3–5 weeks, which is similar to that of nude mice [48]. The loss of function or mutations in the *KRT71* gene may also bring about underdevelopment of the inner root sheath and recessive congenital hypotrichosis in Hereford cattle [49]. Mutations in the *KRT74* gene (c.373delC) could lead to hair thinning and hair loss [50]. In addition, mutations in the *KRT26* gene (A559T) are closely related to the cashmere fineness [51]. Since the number of hair follicles is closely related to the number of hairs and wool fiber diameter, these findings further suggest that KRT71, KRT74, and KRT26 may have a substantial impact on the production of finer and higher-quality wool products. However, despite the fact that the correlation analysis in this study uncovered eight significant results, neither the primary nor the secondary wool fiber diameter achieved statistical significance. This could be regarded as a drawback of the current research. Therefore, it is essential to verify the findings through follow-up research. For example, conducting correlation analyses with a larger number of samples and carrying out polymorphism studies on candidate genes. Through these measures, we may be able to elucidate the potential association between polymorphic sites in candidate genes and wool fineness.

## 5. Conclusions

In the present study, we detected some differentially expressed proteins (KRT26, KRT72, KRT74, KRT71, JUP, XP_012033428.1 and MGST3, etc.), and functional pathway and GO terms (intermediate filaments, keratin I and keratin II, glutathione metabolism, etc.) were found to be closely related to hair follicle development and fineness regulation in fine-wool sheep. These proteins could be used as key candidate factors for hair follicle development and fineness regulation of Gansu alpine fine-wool sheep in the later stage of fine-wool sheep breeding.

## Figures and Tables

**Table 1:** Total protein quality control.

Sample	Protein Concentration (μg/μL)	Total Protein (μg)	Protein Fluid Volume (μL)
F1	0.18	30.46	170.00
F2	0.21	35.02	170.00
F3	0.25	42.50	170.00
F4	0.21	36.04	170.00
C1	0.36	61.88	170.00
C2	0.19	32.30	170.00
C3	0.18	31.01	170.00
C4	0.18	31.28	170.00

**Table 2:** Enrichment results of proteins related to the regulation of hair follicle development and wool fineness in Gansu alpine fine-wool sheep.

Term/Pathway	Protein
GO:0005882 Intermediate filament	KRT26, KRT72, KRT74, KRT71, JUP
CL25737 Intermediate filament protein, and Nail development	KRT26, KRT72, KRT74, KRT71, DNASE1L2
CL25742 Keratin, type I, and Keratin, type II	KRT26, KRT72, KRT74, KRT71
ko00480 Glutathione metabolism	XP_012033428.1, MGST3
ko00190 Oxidative phosphorylation	NDUFB7, NDUFS4
ko04974 Protein digestion and absorption	KLK14
ko00280 Valine, leucine and isoleucine degradation	DBT
ko04024 cAMP signaling pathway	RAP1B, PPP1CB

**Table 3:** Information on keratin and keratin-associated proteins in wool proteomic techniques of different fineness.

Classifications	Protein Name			
Keratin-associated protein	KRTAP6-1	LOC101106296	LOC114110489	LOC101106558
	LOC101103772	LOC101116371	LOC114113348	LOC114113904
	LOC101104027	LOC101116882	LOC114113380	LOC101104203
	LOC101106046	LOC114110486	LOC114113396	LOC114118004
	LOC101107617			
Keratin	KRT25	KRT82	KRT8	KRT5
	KRT27	LOC101112716	KRT78	LOC101111178
	KRTAP4.3	KRT24	KRT6A	LOC114113976
	KRT2.11	KRT26	LOC101111440	LOC101112555
	KRT33A	KRT28	KRT12	LOC101112805
	LOC100526780	KRT20	KRT39	LOC100526784
	LOC100526782	KRT23	KRT13	LOC101112469
	KRT71	KRT40	LOC101111791	KRT80
	KRT17	LOC101112657	KRT1	KRT35
	KRTCAP2	LOC100526781	LOC100141295	LOC101118712
	KRT2	KRT32	KRT14	LOC101118712
	LOC101109951	KRT36	KRT18	KRT19
	LOC101110219	KRT15	KRT3	LOC105610157
	KRT74	KRTDAP	KRT4	LOC101115571
	KRT84	KRT9	KRT79	KRT77

## Data Availability

Proteome data have been deposited to the iProX (Available online: https://proteomecentral.proteomexchange.org/cgi/GetDataset?ID=PXD065079, upload on 16 June 2025), with the PXD accession PXD065079.
